# Work and Mental Distress among Nurses in the Amazon Region of Brazil during the COVID-19 Pandemic

**DOI:** 10.1590/0034-7167-2022-0792

**Published:** 2023-12-04

**Authors:** Darci Francisco dos Santos, Marina Nolli Bittencourt, Priscila Maria Marcheti, José Luís da Cunha Pena, Suellen Cristina da Silva Chaves, Angélica Martins de Souza Gonçalves, Alessandra Matheus Domingos, Maria do Perpétuo do Socorro de Sousa Nóbrega

**Affiliations:** I Universidade Federal do Mato Grosso. Cuiabá, Mato Grosso, Brazil; II Universidade Federal do Mato Grosso do Sul. Campo Grande, Mato Grosso do Sul, Brazil; III Universidade Federal do Amapá. Macapá, Amapá, Brazil; IV Universidade de São Paulo. São Paulo, São Paulo, Brazil; V Universidade Federal de São Carlos. São Carlos, São Paulo, Brazil

**Keywords:** COVID-19, Mental Health, Nursing, Amazonian Ecosystem, Psychic Symptoms, COVID-19, Salud Mental, Ecosistema Amazónico, Enfermería, Síntomas Psíquicos, COVID-19, Saúde Mental, Enfermagem, Ecossistema Amazônico, Sintomas Psicopatológicos

## Abstract

**Objective:**

To assess the relationship between psychopathological symptoms and the social, clinical, and occupational profile of nurses in the Amazon region of Brazil during the COVID-19 pandemic.

**Methods:**

A descriptive cross-sectional online study was conducted in 2020 with 261 nurses. The Symptom Assessment Scale-40 was utilized.

**Results:**

The presence of pre-existing conditions before the pandemic was associated with psychotism (p=0.044). Experiencing constraints and/or violence at work during the pandemic was associated with somatization (p=0.025), and working hours were associated with anxiety (p=0.025). Nurses predominantly exhibited symptoms related to fear (33.7%), tension (34.5%), and a sense that something is wrong in the mind (22.6%).

**Conclusions:**

A significant association was observed between working hours and anxiety symptoms, the experience of constraints and/or violence at work during the COVID-19 pandemic and somatization symptoms, as well as between pre-existing conditions and psychotism symptoms related to COVID-19.

## RESUMO

**Objetivo:**

Avaliar a relação entre sintomas psicopatológicos e o perfil social, clínico e laboral dos enfermeiros da região Amazônica do Brasil durante a pandemia de COVID-19.

**Métodos:**

Realizou-se um estudo descritivo de corte transversal online com 261 enfermeiros em 2020. Utilizou-se a Escala de Avaliação de Sintomas-40.

**Resultados:**

A presença de doenças preexistentes à pandemia associou-se ao psicoticismo (p=0,044). Ter sofrido constrangimentos e/ou violências no trabalho durante a pandemia associou-se à somatização (p=0,025), e a carga horária de trabalho associou-se à ansiedade (p=0,025). Os enfermeiros manifestaram principalmente sintomas relacionados ao medo (33,7%), tensão (34,5%) e sensação de que algo está errado na mente (22,6%).

**Conclusões:**

Foi observada uma associação significativa entre a carga horária de trabalho e sintomas de ansiedade, o sofrimento de constrangimentos e/ou violências durante o trabalho no contexto da pandemia de COVID-19 e sintomas de somatização, bem como entre doenças preexistentes à COVID-19 e sintomas de psicoticismo.

## RESUMEN

**Objetivo:**

Evaluar la relación entre los síntomas psicopatológicos y el perfil social, clínico y ocupacional de las enfermeras en la región amazónica de Brasil durante la pandemia de COVID-19.

**Métodos:**

Se llevó a cabo un estudio descriptivo transversal en línea en 2020 con la participación de 261 enfermeras. Se utilizó la Escala de Evaluación de Síntomas-40.

**Resultados:**

Se encontró una asociación significativa entre la presencia de condiciones preexistentes antes de la pandemia y el psicoticismo (p=0,044). Experimentar restricciones y/o violencia en el trabajo durante la pandemia se asoció con la somatización (p=0,025), y las horas de trabajo se relacionaron con la ansiedad (p=0,025). En general, las enfermeras presentaron predominantemente síntomas relacionados con el miedo (33,7%), la tensión (34,5%) y la sensación de que algo no está bien en la mente (22,6%).

**Conclusiones:**

Se observó una asociación significativa entre las horas de trabajo y los síntomas de ansiedad, así como entre la experiencia de restricciones y/o violencia laboral durante la pandemia de COVID-19 y los síntomas de somatización. Además, se encontró una asociación entre las condiciones preexistentes y los síntomas de psicoticismo relacionados con COVID-19.

## INTRODUCTION

In the Amazon region, factors such as inequality, social vulnerability, precariousness of the healthcare system, and the historical absence of effective public policies, combined with the COVID-19 pandemic, have led to a profound public health crisis, resulting in high mortality rates and overwhelmed healthcare services ^(1-3)^.

Nursing professionals on the frontlines of COVID-19 represent a high-risk group for infection and severe psychological distress ^(4)^. According to the Brazilian Federal Nursing Council (COFEN), from the beginning of the pandemic until December 6, 2022, the Amazon region reported 5,722 cases out of a total of 30,502,501 cases in the country, with 243 deaths out of a total of 872 cases ^(5)^.

Previous studies on the mental distress of nursing professionals during the pandemic have shown that this new reality is associated with increased psychopathological symptoms and critical levels of distress, related to age group, high workload, violence, and lack of psychological support in the workplace ^(6-7)^.

Although they are important references, both primarily depict the reality of nursing professionals in the Southeast region of the country, highlighting the need for studies that reveal the perspective of professionals working in a vulnerable region with low technological density in terms of public health, as is the case in the Amazon region, which was the first to experience collapse and has a small number of professionals, representing only 8% (54,380) of the total number of nurses in Brazil (678,146) ^(8)^.

In the context of the pandemic, the urgency in carrying out work, sometimes under precarious conditions, has led to inattentiveness, difficulty in decision-making, and impacts on the overall well-being of these workers ^(9)^. Tracking psychopathological symptoms expands the mapping of emotional demands among Brazilian nurses and supports the development of institutional support proposals.

## OBJECTIVE

To assess the relationship between psychopathological symptoms and the social, clinical, and occupational characteristics of nurses in the Amazon region of Brazil during the COVID-19 pandemic.

## METHODS

### Ethical considerations

The study adhered to Resolution 466/12 and received approval from the National Research Ethics Committee of Brazil. Prior to their participation, all respondents provided online informed consent.

### Study design, period, and location

This study follows a descriptive cross-sectional design in accordance with the STROBE (Strengthening the Reporting of Observational Studies in Epidemiology) guidelines ^(10)^. Data were collected between April 22 and November 28, 2020, which coincided with the initial wave of COVID-19 in Brazil.

### Population or sample, eligibility criteria

A non-probabilistic sampling approach was employed, utilizing the snowball technique^(11)^. A total of 261 nurses from the Amazon region of Brazil completed a Google® questionnaire, which was widely distributed through various social media platforms. Inclusion criteria required participants to be actively engaged in the nursing profession at any level of healthcare, encompassing both direct patient care and administrative/managerial roles during the COVID-19 pandemic. To ensure the reliability of the study, several measures were implemented, including the collection of email addresses to prevent duplicate responses, exclusion of incomplete or partially filled questionnaires, preservation of the most recent form submitted by each participant, and data extraction and organization under the supervision of four researchers.

### Study Protocol

The questionnaire consisted of 44 questions related to sociodemographic, occupational, and health data, which had previously been validated and underwent a pre-test conducted by 13 doctoral professors. The variables included age, gender, race, marital status, monthly income, weekly working hours, pre-existing medical conditions, professional practice, nature of the workplace institution, duration of education/professional practice, employment status, direct involvement in patient care, experience of suffering due to constraints and/or violence at work, and receipt and type of psychological/emotional support from the workplace institution.

The Symptom Checklist-40 (SCL-40), adapted and validated in Brazil, was used to assess psychopathological symptoms^(12)^. This scale measures self-reported symptoms experienced in the last seven to fifteen days. It was selected due to its good internal consistency (Cronbach’s alpha between 0.73 and 0.88) and regular temporal stability between 7 and 15 days (ranging from 0.40 to 0.82). The scale consists of four subscales, each with 10 items: 1. Anxiety, 2. Somatization, 3. Obsessiveness/Compulsiveness, and 4. Psychoticism. The response format follows a Likert-type scale with three levels of intensity: 0 = no symptom, 1 = mild symptom, and 2 = severe symptom. The raw score is calculated by summing the values of 0 to 2 for each item and dividing it by the number of items in each dimension. In case a respondent does not answer any item on the scale, the division is performed by the number of items answered ^(12)^.

### Data Analysis and Statistics

The data were tabulated using Excel 2016 software, and subsequent statistical tests were conducted using IBM SPSS version 25. Absolute and relative frequencies, means, and standard deviations (SD) were calculated. The Wilcoxon-Mann-Whitney test and Kruskal-Wallis test (F test used in ANOVA) were applied, and univariate and bivariate analyses were conducted to compare the domains of SCL-40-R with the social and clinical profile. A 95% confidence interval and a significance level of 5% were used for all tests (p ≤ 0.05). The internal consistency of the EAS-40 was assessed by calculating Cronbach’s alpha coefficient.

## RESULTS

### Sample Characteristics

The sample comprised 261 nurses from the states in the Amazon region of Brazil. Among them, 220 (84.3%) were female, with ages ranging from 20 to 39 years. In terms of marital status, 159 (60.9%) were married or in a stable relationship. Regarding racial background, 132 (50.6%) identified as mixed race, 83 (31.8%) as white, and 39 (14.9%) as black. With regards to monthly income, 14 (5.4%) earned less than one minimum wage, 118 (45.3%) earned between one to three minimum wages, and 74 (28.4%) earned between four to six minimum wages (Minimum wage at the time of data collection: R$ 1,100.00 = U$ 231.46). The majority of professionals, 159 (60.9%), were the primary caregivers or financial providers in their households. The majority of participants fell under the category of formal workers, with a weekly working hour of at least 40 hours or more, 158 (60.6%), working as public servants, 164 (62.8%), and being directly involved in patient care, 145 (55.6%).

Regarding the clinical profile, the majority did not have any pre-existing clinical conditions prior to the pandemic, 184 (70.5%), while 77 (29.5%) did. Among those with pre-existing conditions, one in ten did not receive any treatment, 10 (13%). In terms of psychological follow-up and/or psychiatric treatment, 38 (14.6%) and 36 (13.8%) had undergone such care before the pandemic, respectively, and 18 (6.9%) were taking psychiatric medication without a medical prescription. The majority, 195 (74.7%), did not stay in a location without contact with their families, and 231 (88.5%) had friends, family members, and colleagues who had been infected with COVID-19, while 145 (55.6%) had experienced the loss of friends, family members, or colleagues due to the disease.

### Presentation of Psychopathological Symptoms

The psychopathological symptoms are presented in Table 01, indicating that nurses in the past 14 days reported high levels of concern regarding specific symptoms. These include “feeling fearful” (88, 33.7%), “muscle aches (body pain)” (87, 33.3%), “feeling tense or stuck” (90, 34.5%), and “having the idea that something is wrong with their mind” (59, 22.6%).

**Table 1:** Characterization of the Symptom Checklist-40-R (SCL-40-R) responses among nurses from the Amazon region of Brazil, N: 261, Brazil, 2020

	Extremely Concerned	Not Concerned	Slightly Concerned
	n(%)	n(%)	n(%)
Psychoticism			
Weakness or dizziness	51(19.5)	127(48.7)	83(31.8)
Heart or chest pain	55(21.1)	126(48.3)	80(30.7)
Fear in open spaces or on the streets	74(28.4)	105(40.2)	82(31.4)
Suicidal thoughts	10(3.8)	222(85.1)	29(11.1)
Sudden unexplained fear	58(22.2)	122(46.7)	81(31.0)
Fear of going out alone	34(13.0)	163(62.5)	64(24.5)
Back and hip pain	82(31.4)	94(36.0)	85(32.6)
Feeling of insignificance	60(23.0)	134(51.3)	67(25.7)
Fear	88(33.7)	69(26.4)	104(39.8)
Nausea. queasiness. or upset stomach	59(22.6)	130(49.8)	72(27.6)
Obsessive-Compulsivity			
Muscle aches (body pain)	87(33.3)	76(29.1)	98(37.5)
Feeling observed and subject to comments by others	32(12.3)	171(65.5)	58(22.2)
Fear of taking buses. subways. or trains	65(24.9)	135(51.7)	61(23.4)
Difficulty breathing	40(15.3)	135(51.7)	86(33.0)
Episodes of hot or cold sensations	42(16.1)	138(52.9)	81(31.0)
Need to avoid certain things. places. or activities that trigger fear	75(28.7)	113(43.3)	73(28.0)
Mental lapses or momentary inability to think or recall information	70(26.8)	110(42.1)	81(31.0)
Numbness or tingling in specific body parts	44(16.9)	157(60.2)	60(23.0)
Sense of hopelessness regarding the future	58(22.2)	102(39.1)	101(38.7)
Somatization			
Difficulty concentrating	82(31.4)	78(29.9)	101(38.7)
Feeling weakness in certain body parts	46(17.6)	125(47.9)	90(34.5)
Feeling tense or stuck	90(34.5)	81(31.0)	90(34.5)
Experiencing heaviness in the arms and legs	53(20.3)	136(52.1)	72(27.6)
Feeling uncomfortable when being observed or talked about by others	46(17.6)	153(58.6)	62(23.8)
Having to repeat the same actions. such as touching. counting. or washing	56(21.5)	125(47.9)	80(30.7)
Having urges to break or destroy things	25(9.6)	189(72.4)	47(18.0)
Feeling excessively self-conscious or overly concerned about others	60(23.0)	122(46.7)	79(30.3)
Feeling that everything requires an excessive amount of effort	54(20.7)	123(47.1)	84(32.2)
Anxiety			
Waves of terror or panic	36(13.8)	164(62.8)	61(23.4)
Frequently getting involved in arguments	26(10.0)	178(68.2)	57(21.8)
Feeling nervous when left alone	28(10.7)	176(67.4)	57(21.8)
Feeling lonely even in the company of others	41(15.7)	136(52.1)	84(32.2)
Feeling excessively restless and unable to stay still	44(16.9)	152(58.2)	65(24.9)
Engaging in spinning or throwing objects	16(6.1)	212(81.2)	33(12.6)
Fear of fainting in public	14(5.4)	216(82.8)	31(11.9)
Persistent sense of detachment from others	23(8.8)	183(70.1)	55(21.1)
Feelings of guilt	46(17.6)	157(60.2)	58(22.2)
Belief that something is wrong with your mind	59(22.6)	143(54.8)	59(22.6)

Table 2 presents a detailed analysis of the internal consistency of the EAS-40 scale, examining each item individually. The results indicate that all four domains exhibited coefficients higher than 0.70 and 0.76, respectively. Moreover, the scores for the individual items fell within the range of 0.6 to 0.7.

**Table 2:** Detailed analysis of the internal consistency of the EAS-40 items, responded by nurses from the Amazon region of Brazil, N: 261, Brazil, 2020

	Mean	Standard Deviation	Cronbach's Alpha if the item is excluded
Psychoticism			
Weakness or dizziness	2.123	0.707	0.707
Heart or chest pain	2.096	0.714	0.702
Feeling fear in open spaces or on the streets	2.031	0.774	0.695
Thoughts of suicide	2.073	0.380	0.716
Sudden onset of fear without reason	2.088	0.726	0.675
Fear of going out alone	2.115	0.603	0.696
Back and hip pain	2.011	0.801	0.662
Feeling of insignificance	2.027	0.698	0.692
Feeling afraid	2.061	0.857	0.686
Nausea. queasiness. or upset stomach	2.050	0.708	0.696
Obsessive-Compulsivity			
Muscle soreness (body pain)	2.042	0.842	0.689
Feeling under surveillance and subject to scrutiny by others	2.100	0.580	0.685
Compulsive checking and rechecking of tasks	2.192	0.719	0.690
Fear of using buses. subways. or trains	1.985	0.696	0.695
Difficulty breathing	2.176	0.673	0.690
Episodes of sudden heat or cold sensations	2.149	0.671	0.672
Necessity to avoid specific things. places. or activities that provoke fear	1.992	0.754	0.684
Temporary cognitive lapses or memory lapses	2.042	0.761	0.702
Numbness or tingling sensations in body parts	2.061	0.629	0.686
Experiencing a sense of hopelessness regarding the future	2.165	0.764	0.708
Somatization			
Difficulty concentrating	2.073	0.836	0.701
Feeling weakness in certain body parts	2.169	0.703	0.689
Feeling tense or trapped	2.000	0.832	0.684
Experiencing heaviness in the arms and legs	2.073	0.690	0.701
Feeling uncomfortable when being observed or talked about by others	2.061	0.642	0.712
Having to repeat the same actions. such as touching. counting. or washing	2.092	0.717	0.726
Having urges to break or destroy objects	2.084	0.519	0.735
Feeling excessively self-conscious or concerned about others	2.073	0.728	0.715
Feeling restless in crowded places. while shopping. or at the cinema	1.989	0.731	0.717
Feeling that everything requires excessive effort	2.115	0.719	0.706
Anxiety			
Feeling nervous when left alone	2.111	0.561	0.734
Feeling lonely even when surrounded by others	2.165	0.673	0.750
Feeling restless to the point of being unable to sit still	2.080	0.642	0.745
Engaging in spinning or throwing objects	2.065	0.429	0.758
Fear of fainting in public	2.065	0.411	0.767
Persistent sense of emotional distance from others	2.123	0.534	0.742
Experiencing feelings of guilt	2.046	0.631	0.730
Belief that something is wrong with your mind	2.000	0.674	0.727

Table 3 displays the findings concerning the psychopathological symptoms of SCL-40-R alongside sociodemographic and work-related variables. A significant correlation was observed between the variable “presence of pre-existing diseases before the pandemic” and the Psychoticism domain (p=0.044). Furthermore, the variable “experienced constraints and/or violence in the course of their work since the beginning of the COVID-19 pandemic” exhibited a significant association with the Somatization domain (p=0.025). Moreover, the variable “workload hours” demonstrated a significant association with the Anxiety domain (p=0.025).

**Table 3:** Comparative analysis of SCL-40-R scores and sociodemographic, clinical, and laboratory data of nurses from the Amazon region of Brazil, N: 261, Brazil, 2020

Variable	Psychoticism		Obsessive-Compulsivity		Somatization		Anxiety	
	Mean ± SD	*p* value	Mean ± SD	*p* value	Mean ± SD	*p* value	Mean ± SD	*p* value
Age Group		0.829^2^		0.469^2^		0.642^2^		0.496^2^
20-39 years	2.05±0.39		2.08±0.37		2.07±0.35		2.07±0.34	
40-59 years	2.09±0.36		2.11±0.39		2.11±0.35		2.11±0.32	
≥60 years	2.20±0.26		1.93±0.15		2.05±0.09		2.00±0.00	
Gender		0.391^1^		0.391^1^		0.810^1^		0.206^1^
Female	2.06±0.37		2.08±0.38		2.08±0.35		2.08±0.33	
Male	2.10±0.38		2.12±0.34		2.08±0.31		2.01±0.31	
Total Monthly Income		0.127^2^		0.915^2^			0.833^2^	0.732^2^
< 1 minimum wage (MW)	2.12±0.31		2.06±0.17		2.09±0.25		2.08±0.18	
1-3 MW	2.02±0.39		2.08±0.38		2.06±0.36		2.06±0.32	
4-6 MW	2.06±0.37		2.09±0.38		2.09±0.34		2.07±0.36	
7-9 MW	2.13±0.35		2.05±0.45		2.05±0.39		2.06±0.33	
≥ 10 MW	2.24±0.33		2.20±0.34		2.18±0.29		2.19±0.30	
Pre-existing Diseases		0.044^1^		0.104^1^			0.536^1^	0.298^1^
Yes	1.98±0.42		2.02±0.44		2.04±0.41		2.02±0.37	
No	2.10±0.35		2.12±0.34		2.10±0.31		2.11±0.31	
Weekly Workload		0.411^2^		0.664^2^			0.076^2^	0.052^2^
Over 44 hours per week	2.01±0.41		2.00±0.45		1.96±0.42		1.98±0.46	
40 hours per week	2.10±0.37		2.12±0.37		2.11±0.34		2.09±0.31	
20 hours per week	2.11±0.29		2.11±0.31		2.13±0.34		2.03±0.33	
36 hours per week	2.03±0.30		2.08±0.34		2.11±0.25		2.17±0.22	
Weekly Workload								
44 hours per week	2.23±0.53		2.19±0.40		2.17±0.43		2.11±0.36	
Not applicable	2.08±0.41		2.12±0.34		2.11±0.34		2.12±0.22	
Experienced constraints and/or violence in the workplace during the COVID-19 pandemic		0.596^2^		0.286^2^		0.025^2^		0.279^2^
Yes	2.03±0.44		2.02±0.46		2.00±0.41		2.05±0.42	
Not	2.10±0.31		2.12±0.33		2.12±0.30		2.10±0.26	
Not applicable	2.03±0.45		2.15±0.32		2.13±0.32		2.14±0.28	
Received psychological/emotional support from their workplace during the COVID-19 context		0.218^1^		0.578^1^		0.207^1^		0.656^1^
Yes	2.15±0.29		2.13±0.31		2.15±0.29		2.14±0.27	
No	2.05±0.39		2.08±0.39		2.07±0.35		2.08±0.34	

1 Mann-Whitney U test;

2 Kruskal-Wallis test.

## DISCUSSION

This study was conducted among married nurses of mixed race, aged between 20 and 39 years, with a monthly income of up to three times the minimum wage, and working 40 hours or more per week in the Brazilian Amazon region. This region has been severely affected by the pandemic, with three of its states experiencing a higher incidence of COVID-19 cases, making it a focal point of the public health crisis due to social inequality, lack of effective public policies, and limited resources to address the needs of those affected by the disease ^(1-2,13)^.

It is important to note that no significant correlation was found between age, race, marital status, or monthly income and psychopathological symptoms. However, the observed predominance in terms of age, gender, and marital status is consistent with previous studies conducted, including those during the pandemic ^(14-17)^. The results related to marital status shed light on the challenges faced by these professionals in balancing work and family responsibilities, including concerns about transmitting the disease to their loved ones. This situation can lead to family strain and have a psychological impact on these healthcare professionals ^(15)^.

In contrast to another study on nursing professionals ^(18)^, this research yielded different findings regarding race, with a more diverse representation and less dominance of white nurses. Regarding the work schedule, it was observed that 60.6% of the participants work 40 hours per week or more. These findings align with the existing literature, which highlights the common occurrence of nurses having double shifts, resulting in less time for family, leisure activities, and increased physical and mental strain ^(17,19)^.

An important aspect to consider is that 159 (60.9%) of the participants are responsible for caring for their families and ensuring financial stability. This, combined with societal gender expectations and limited time for family, can contribute to job dissatisfaction ^(14,20-21)^. Furthermore, being the primary provider also brings about fear and uncertainty, particularly during the pandemic, which can impact the mental health of these professionals and the quality of care they provide ^(20-21)^.

It is worth noting that when these professionals need to take time off due to their own distress, it often results in staffing shortages, placing additional burden on their colleagues. This, in turn, makes the work schedule more demanding for their colleagues, leading to dissatisfaction with the work environment ^(21)^.

In this study, it is noteworthy that all domains of the EAS-40 scale consistently showed high scores, irrespective of gender, when compared to a previous study ^(12)^. Specifically, in relation to the Psychoticism domain, nurses with pre-existing clinical conditions were more likely to exhibit psychotic symptoms. The authors suggest that the nursing profession itself entails risk factors for chronic diseases, which, when combined with fatigue and occupational stress, limit time available for self-care activities and contribute to the development of both physical and psychopathological symptoms ^(16)^.

It is hypothesized that the significant correlation between pre-existing clinical conditions and scores in the Psychoticism domain can be attributed to previous studies ^(16,22)^. The nursing profession is associated with risk factors for developing chronic diseases, such as obesity and smoking, which are precursors to these conditions. Additionally, the combination of fatigue and occupational stress leaves nurses with limited time to prioritize their own health, resulting in symptoms such as burnout and psychoticism.

Furthermore, within the context of the pandemic, nursing professionals have been confronted with unsatisfactory working conditions in healthcare settings, including inadequate access to Personal Protective Equipment (PPE) and instances of verbal, physical, and psychological abuse. These circumstances have caused physical, psychological, and moral harm, as well as discrimination and prejudice from certain segments of society ^(23-25)^.

In the Psychoticism domain, nurses grapple with physical issues such as back and hip pain (82, 31.4%), as well as emotional aspects like fear (88, 33.7%), which intensify the mental distress experienced by these professionals. While these symptoms are present in a portion of the sample, thoughts of self-harm (222, 85.1%), feelings of worthlessness (134, 51.3%), and fear of venturing outside alone (163, 62.5%) indicate attempts to achieve a reasonable biopsychosocial equilibrium that nurses strive for amidst challenging circumstances. This suggests that these professionals endeavor to minimize the risks of illness.

Regarding the Obsessive-Compulsive domain, a significant correlation was identified between the variables in the study. Nurses encounter concerns related to muscular pain (185, 70.8%) and a sense of hopelessness regarding the future (159, 60.9%).

Another noteworthy finding of the study is the significant link between experiencing constraints and/or violence in the course of work and scores in the Somatization domain. It is believed that these nurses have been adversely affected by the violence inflicted by society, stemming from the fear experienced, the infodemic, and the dissemination of misinformation regarding modes of disease transmission. These factors have detrimental effects on the mental health of nurses ^(26)^. Violence generates anger, suffering, feelings of guilt, worry, tension, stress, despair, rage, fear, sadness, frustration, and disinterest in working in the field. Over time, these emotions have negative repercussions on the mental well-being of nurses and contribute to a decrease in the number of professionals ^(25)^. The somatization of the negative effects of the work environment can give rise to various issues, including musculoskeletal disorders ^(27,29)^, and a decline in the quality of healthcare due to professionals’ feelings of dissatisfaction and fear ^(27-28,30)^.

In the Somatization domain, nurses struggle with difficulty concentrating (82, 31.4%) and feel tense (90, 34.5%). Considering these manifestations in their daily lives and the high workload (158, 60.6%), they face the imminent risk of making errors in the nursing work process. At the same time, they may feel physically well when they don’t experience weakness in certain body parts (125, 47.9%) and don’t feel heaviness in their arms and legs (136, 52.1%). Emotionally, they don’t feel uncomfortable when people observe or talk about them (153, 58.6%) and don’t have a desire to break or destroy things (189, 72.4%). These aspects may indicate an intrinsic resistance profile to work but are also influenced by the demands of the current situation they are facing, as they attempt to be resilient.

A significant correlation between the Anxiety domain score and the variable of workload stood out in this study. Research shows that the intense workload involved in caring for COVID-19 patients contributes to the psychological distress of nurses ^(31-32)^, and the longer the years of service, the less rest and family interaction, leading to physical and mental stress ^(15)^.

In the Anxiety domain, nurses face the workload, which results in concerns manifested as waves of terror or panic (97, 37.2%) and feelings of loneliness (125, 47.9%) and that something is wrong with their mind (118, 45.2%), due to the emergent situation in the Amazon region. Considering that the majority of the sample in this study consists of married nurses who work more than 40 hours per week and likely face demands related to supporting their families, as well as the fear of being disease vectors ^(33)^, the obligation to provide quality care, along with the inherent responsibilities of the profession and long-term precarious work ^(34)^, combined with work overload, contribute to the emotional imbalance of these professionals ^(35)^.

However, it is necessary to criticize the regional collapse, the precariousness of the healthcare system, and the political handling of the pandemic in the Amazon region, conditions that have demanded so much from nurses to the point of physical and emotional exhaustion, fear, and panic while caring for seriously ill individuals, often including their own family members and colleagues, without personal protective equipment. Additionally, they had to deal with their own mental distress in the absence of psychological support.

In this regard, studies highlight the importance of providing support, crisis management, and creating psychological support teams, including in-person or online psychosocial interventions, which contribute to alleviating the psychological distress of nurses. In the workplace, measures that allow for the reorganization of the work process, such as adopting shorter shifts, separating teams between caregivers and non-caregivers of COVID-19 patients, and training professionals for work standardization, are potential strategies ^(32,35)^.

### Limitations of the Study

The study has several limitations that should be acknowledged. Firstly, the sample used was non-probabilistic and relied on the snowball technique for participant recruitment, which may limit the generalizability of the results. However, it is important to recognize that given the constraints of social distancing measures, this was the most practical approach for collecting data on the mental health of nurses in the region. Additionally, the predominance of female participants in the sample prevents the findings from being fully generalizable to the broader population.

### Contributions to the Field

Nurses in the Amazon region have played a critical role in delivering healthcare services during the height of the COVID-19 pandemic. The findings of this study have important implications for supporting this professional group not only in the current pandemic context but also in the future. It is recommended that further research be conducted, encompassing a larger and more diverse sample of nurses from the Amazon region, to gain a deeper understanding of the mental health impacts in a setting where economic and political investments are yet to bring about substantial changes.

## CONCLUSIONS

The COVID-19 pandemic has had a direct impact on the physical and mental well-being of nurses in the Amazon region. Significant associations have been observed between workload and anxiety symptoms, experiences of constraints and/or violence during work in the context of the pandemic, and somatization symptoms. Similarly, a link has been found between pre-existing conditions and symptoms of psychoticism. These findings underscore the need for targeted interventions and support mechanisms to address the mental health challenges faced by nurses in this region.
